# Dr. Thomas Nash Reed

**Published:** 1870-09

**Authors:** 


### OBITUARY.

*Dr. Thomas Nash Reed.*—With deep regret we announce the death of Dr. Reed of Danville Va. by the rail road accident last month near the Green Briar White Sulphur Springs.

Dr. Reed was a native of Charlotte Co., Va., was educated at Hampden Sydney College, and a graduate of the Baltimore College of Dental Surgery of the Class of 1866. He was a gentleman of fine scholastic attainments, and possessed a very remarkable degree of mechanical ingenuity and inventive faculty. The intercourse of the Faculty of the Baltimore College of Dental Surgery, with Dr. Reed led them to think highly of his integrity and moral worth. Under a modest and unassuming exterior, he concealed abilities which would have given the profession reason one day to be proud of him.

